# Home Healthcare in South Korea: A Literature Review on Access, Quality, and Cost

**DOI:** 10.34172/ijhpm.9207

**Published:** 2026-04-06

**Authors:** Dae Hyun Kim, Douglas A. Jones, Claudia Guerrazzi Young, Radhika Amin

**Affiliations:** ^1^Department of Health Management Policy, Georgetown University, Washington, DC, USA.; ^2^Department of Business Administration, Auburn University at Mongomery, Montgomery, AL, USA.; ^3^Massey College of Business, Belmont University, Nashville, TN, USA.; ^4^School of Medicine, Georgetown University, Washington, DC, USA.

**Keywords:** South Korea, Home Health, Access, Quality, Cost

## Abstract

**Background:**

South Korea faces significant healthcare challenges due to its rapidly aging population, necessitating alternative care models to hospital-based systems. In response, home healthcare has emerged as a promising strategy to support aging in place while maintaining care continuity and system sustainability.

**Methods:**

This literature review examines home healthcare in South Korea through the lens of healthcare access, quality, and cost, which are the three dimensions of the "Iron Triangle." Online databases were used to identify papers published from 2010 to 2025, from which 39 documents were selected which examine the impact of home healthcare in South Korea, specifically in terms of access, quality, or cost.

**Results:**

Results indicate that home healthcare effectively addresses healthcare access disparities, especially for elderly and disabled populations, by overcoming geographic and mobility barriers through integrated care models and remote technologies. Quality of care is enhanced through patient-centered approaches, multidisciplinary collaboration, and proactive home visits, leading to improved clinical outcomes and higher patient satisfaction. Economically, home healthcare demonstrates potential cost savings by reducing hospitalizations and emergency care usage, particularly for patients with chronic conditions and high utilization patterns.

**Conclusion:**

Sustainability remains contingent upon effective reimbursement and resource allocation policies. Policy implications include expanding healthcare infrastructure, investing in caregiver training, adopting advanced technologies to maximize the effectiveness of home healthcare, and promoting a sustainable and equitable healthcare system in South Korea. Strategic integration of home healthcare within national insurance and long-term care frameworks will be essential to realizing its full system-level benefits.

## Background

**Highlights**

- Home healthcare reduces barriers, enhancing elderly health access in South Korea.
- Multidisciplinary home care improves clinical outcomes and patient satisfaction.
- Structured home care programs significantly lower healthcare system costs.
- Effective policies are crucial for global home healthcare scalability.

South Korea’s healthcare system has long been recognized for its universal coverage, technological advancements, and strong hospital-based infrastructure.^1^ The most notable program, the long-term care insurance (LTCI) program, launched in 2008, provides universal coverage for elderly and younger individuals with disabilities needing help with daily activities, offering home or institutional care and devices.^1^ However, as the nation faces a rapidly aging population, the limitations of its institutional care model have become increasingly evident. The percentage of South Koreans aged 65 and older is projected to reach 40% by 2050, posing significant challenges for the healthcare system’s sustainability and capacity to provide adequate care.^1^ Traditional hospital-based care is becoming less viable due to rising costs, overburdened acute care facilities, and the growing preference among elderly individuals to age at home.^2^ In this context, home healthcare has emerged as a potential solution to improve access to medical services, maintain quality of care, and control health expenditures,^3^ which are key dimensions commonly referred to as the “Iron Triangle” of healthcare. However, the development of home health services in South Korea remains in its early stages, with substantial barriers to implementation, regulation, and integration with the broader healthcare system.

 Despite a well-established LTCI system, home-based medical care remains underutilized in South Korea compared to other high-income OECD (Organisation for Economic Co-operation and Development) countries,^2^ As of 2020, only 0.4% of medical institutions in South Korea provided home healthcare services, a stark contrast to countries such as Japan and Germany, where home-based care is a standard component of healthcare delivery.^2^ Limited provider participation, regulatory restrictions, and cultural expectations surrounding family caregiving contribute to these gaps in service availability.^4^ Additionally, healthcare access disparities remain a pressing issue, particularly in rural and aging communities where hospital-based services are geographically and financially burdensome.^5^ Home healthcare presents opportunity to bridge these gaps by offering medical services within patients’ homes, reducing travel barriers, and enabling earlier interventions that may prevent costly hospitalizations.^3^

 Ensuring quality of care in home health services is essential to its viability as an alternative to institutional care. While South Korea has made strides in integrating community-based healthcare services, a standardized, patient-centered home health model remains underdeveloped.^6^ Recent pilot programs, such as the Patient-Centered Integrated Model of Home Health Care Services in Korea, highlight the potential for multidisciplinary collaboration between physicians, nurses, and social workers to enhance care coordination.^2^ However, challenges persist in maintaining care continuity, monitoring patient outcomes, and ensuring adequate provider training.^7^

 The economic sustainability of home healthcare in South Korea remains uncertain, as financial incentives for providers and affordability for patients have yet to be fully addressed. Home-based services have the potential to reduce overall healthcare costs by decreasing avoidable hospital admissions and emergency department visits, yet direct labor costs for home health workers present a financial challenge.^8^ Hence, the goal of this study is to examine the current landscape of home healthcare in South Korea, focusing on its impact on healthcare access, quality, and cost. By synthesizing existing research, this study aims to identify key challenges and opportunities in scaling home-based healthcare services within the country’s unique healthcare system. Given the rapid demographic transition and increasing demand for alternatives to hospital-based care, understanding the feasibility and implications of home health expansion is critical for informing future policy and practice.

## Conceptual Framework (Iron Triangle-Access, Cost, and Quality)

 The iron triangle of healthcare^9^ conceptualizes the relationship between three fundamental dimensions of health system performance: access, quality, and cost. The framework suggests that improvements in one dimension often come at the expense of another, given the constraints of finite resources. While access is necessary to ensure equitable healthcare delivery, expanding access without proper management may increase costs or lead to quality deficiencies; similarly, efforts to enhance quality may drive up expenditures. The Iron Triangle has been widely used to assess healthcare delivery models, including home healthcare, which is often positioned as a lower-cost alternative to institutional care while potentially improving access and quality.^10^

 Access, in this framework, refers to the ability of individuals to obtain timely and appropriate health services. Andersen’s behavioral model of health services use emphasizes that access is shaped by both structural and individual-level factors, including the availability of providers, geographic distribution, financial barriers, and patient characteristics.^11^ Home healthcare inherently enhances structural access by delivering medical and supportive services directly to patients, particularly benefiting older adults, individuals with mobility challenges, and those residing in rural or underserved areas.^12^ Studies have found that home health agencies cover nearly all Medicare beneficiaries in the US, suggesting broad theoretical access^13^; however, disparities in actual access persist, as research has shown that racial and socioeconomic inequities limit the ability of disadvantaged populations to obtain high-quality home health services.^14^ This highlights that while home healthcare has the potential to expand access, structural inequities may still restrict high-quality service availability to certain populations.

 Quality in healthcare is multidimensional, encompassing both clinical effectiveness and patient-centered care. The structure-process-outcome model developed by Donabedian provides a foundational framework for understanding quality in home healthcare, emphasizing that high-quality outcomes result from well-structured systems and effective care processes.^15^ Home health services enable personalized, continuous, and relationship-centered care, which can lead to better patient adherence, satisfaction, and clinical outcomes.^16^ However, ensuring consistency and standardization in home-based settings is challenging. Variability in the home environment, caregiver competency, and care coordination can lead to inconsistencies in quality across home health agencies.^11^ Nonetheless, home healthcare has been shown to reduce avoidable hospitalizations and improve chronic disease management when services are well-coordinated.^12^ Systematic efforts, such as value-based purchasing initiatives, aim to incentivize agencies to deliver high-quality care while maintaining efficiency.^11^

 Cost containment is the third dimension of the Iron Triangle and a key driver of home health expansion. Economic theory suggests that shifting care from high-cost institutional settings to lower-cost home-based care should generate savings, provided that care remains effective.^17^ Systematic reviews confirm that home healthcare can be cost-saving or cost-effective in many cases. Curioni and colleagues found that home-based care was cost-saving in 7 out of 14 reviewed studies and cost-effective in 2 additional studie,^18^ demonstrating that home care can often achieve similar or better health outcomes at lower costs compared to hospital-based care. Likewise, the Hospital-at-Home model, which provides hospital-level care in patients’ homes, has shown 19% cost reductions while maintaining or improving health outcomes.^12^ Despite these advantages, financial sustainability remains a concern. Medicare’s home health payments are currently higher than agencies’ actual costs, raising concerns about overpayments and inefficiencies.^13^ Furthermore, out-of-pocket costs for long-term home-based services can be prohibitively high, limiting affordability and posing barriers to access.^19^ Achieving cost efficiency while maintaining equitable access and quality remains a central challenge in home healthcare policy.

 The interdependencies among access, quality, and cost present complex trade-offs and potential synergies in home healthcare. Expanding access to home health services without proper workforce planning and reimbursement structures may reduce the quality of care due to overburdened providers and inconsistent service availability. Conversely, policies that promote high-quality, well-coordinated home health programs have been shown to lower costs by preventing avoidable hospitalizations and improving chronic disease outcomes^10,18^; therefore, within this theoretical framework, it is crucial to identify and implement innovative strategies, such as telehealth, remote monitoring, and integrated care coordination, that can simultaneously enhance access, maintain quality, and contain costs, thereby effectively mitigating the inherent trade-offs outlined by the Iron Triangle.^12^

 Given these theoretical considerations, this study seeks to address the following research questions: (1) How does the current model of home healthcare influence patient access, quality of care, and overall healthcare costs in South Korea? (2) What challenges and barriers exist in scaling home healthcare services within South Korea’s unique healthcare system? (3) What policy and operational strategies can be implemented to optimize the balance among access, quality, and cost in home healthcare in the context of South Korea’s demographic and healthcare shifts?

## Methods

 The literature review followed the Preferred Reporting Items for Systematic Reviews and Meta-Analyses (PRISMA) guidelines to ensure a structured and transparent approach to study selection and evaluation.^20^

###  Eligibility Criteria

 Studies were included if they were published in English, provided empirical evidence or systematic assessments of home healthcare in South Korea, and addressed at least one of the key outcome measures: access, quality, or cost. As the LTCI program was not introduced until 2008, the search syntax employed restrained the searches to 2009 and later with the database search being conducted in February of 2025. Studies that were opinion pieces, editorials, or did not specifically evaluate home healthcare in South Korea were excluded. EndNote 21^21^ was used for reference management, duplicate removal, and document organization. Data extraction focused on identifying key themes related to access, quality, and cost, with findings synthesized to provide a comprehensive understanding of the role of home healthcare in South Korea.

###  Information Sources and Search Strategy

 A comprehensive search was conducted across Academic Search Premier, Business Source Premier, CINAHL, PubMed, and Scopus to identify relevant literature examining the impact of home healthcare in South Korea, specifically in terms of access, quality, or cost assessment or evaluation. Boolean operators were used to refine searches in alignment with the specifications of each database. To confirm the inclusion of relevant literature, the list of periodicals and other publications covered by each database was reviewed.

###  Methodological Quality Assessment

 Methodological quality of all included studies was assessed using the Mixed Methods Appraisal Tool version 2018.^22,23^ The Mixed Methods Appraisal Tool allows the use of a single instrument for the assessment of qualitative, quantitative randomized controlled trials, quantitative non-randomized, quantitative, and mixed methods studies. The tool includes 27 questions, two screening questions which apply to all studies and five questions which apply to each study based upon which of the five methodological approaches was employed by the researchers. Each question was answered, based on information reported in the publications, using one of the following responses: “yes,” “no,” or “can’t tell.” Methodological quality was assessed by three reviewers with inconsistencies resolved through discussion.

###  Data Extraction and Analysis

 Reviewers used a pre-designed data extraction tool to collect the following information from each study included: authors, year of publication, title of article, journal title, methods, objectives, problem statement(s), result(s), limitations, practical application(s), and conclusion(s).

## Results

###  Study Selection

**Figure 1:**
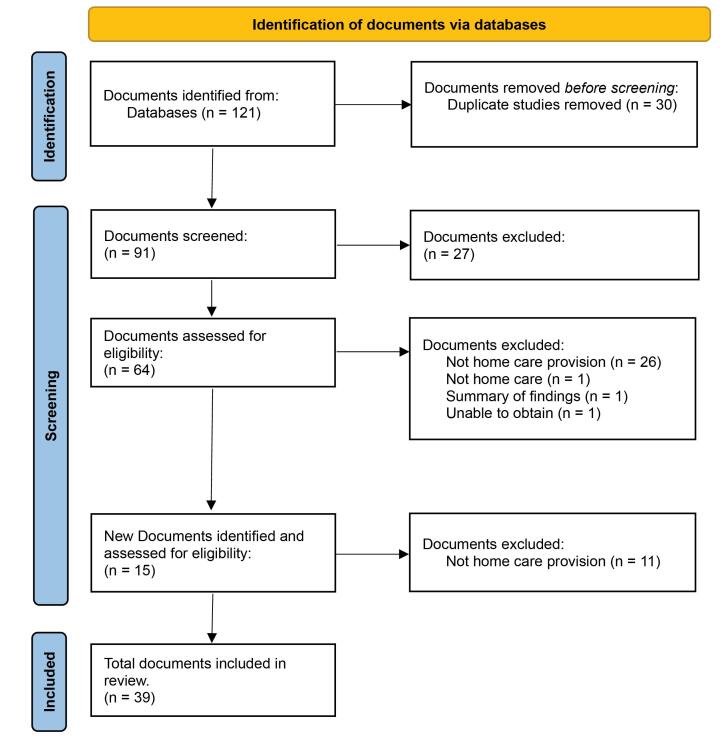
Search Process.

 The initial search of the five databases yielded 121 documents of which 30 were removed as duplicates and three were excluded due to unavailability in English. The remaining 88 documents were screened based on titles, keywords, and abstracts, resulting in the exclusion of 27 documents that did not meet the research focus. Full-text documents were obtained, with the exception of one which was unavailable. To supplement the database search, the snowball method was used to identify 15 additional documents through reference list screening of relevant articles. A total of 75 studies were assessed for potential inclusion, and 39 documents met eligibility criteria. The PRISMA flow diagram illustrating the study selection process is presented in Figure 1.

###  Study Characteristics

 All 39 articles included in the study were published between 2011 and 2025. All articles were published in scholarly journals with approximately 33 percent of articles published in nursing journals. Only three articles provided evidence of the impact of home healthcare services on access while 15 articles provided evidence of the impact of home healthcare services on cost and utilization. Research evaluating the impact of home healthcare on quality made up the majority of the studies with 23 articles providing some evidence. Table 1 summarizes the data for each study specific to dimension. If a study provides evidence for more than one dimension, the findings from the study, specific to the dimension, are included under the findings for that dimension. Additionally, Table 2 synthesizes the core findings of the review.

**Table 1:** Included Studies

**Study**	**Study Design**	**Population Studied**	**Purpose of the Study**	**Findings Specific to Home Healthcare**
**Access**				
Kim et al^31^	Description survey study using a cross-sectional design.	Caregivers of 307 elderly patients receiving long-term care services.	The study investigates how family caregiver presence affects the use of long-term care services.	Family caregivers significantly influence the type of long-term care services used. Older adults with lower physical functioning and more problem behaviors tend to use nursing homes more frequently. The presence of a spouse as a caregiver increased the likelihood of using home healthcare services.
Kim et al^32^	Secondary data analysis.	27 625 receiving home care and community care services.	The research aims to analyze the association between living arrangements and caregiver types with institutionalization in LTCI beneficiaries.	Home care beneficiaries living with non-family members are more likely to be institutionalized within one year compared to those living with spouses. Individuals without a primary caregiver or with a paid caregiver have higher odds of institutionalization than those with a spouse as a caregiver.
Kim et al^33^	Phone-based survey instrument with statistical analyses.	755 people with disabilities aged 20 or greater.	The study aimed to explore the demand for HBPC from the perspectives of people with disabilities and caregivers.	Higher demand for HBPC was noted in those aged ≥65 years and with lower education levels. Home-bound individuals showed a 56.6% demand for HBPC, surpassing non-homebound subjects. Severe disability correlated with a higher demand for HBPC, with 27.8% expressing this need. Proxy-reported respondents had a higher demand for HBPC (39.57%) compared to self-reported respondents (19.49%).
**Quality**				
Myunghee and Ji Young^44^	A non-equivalent control group pre-post-test quasi-experimental study.	46 post spinal surgery patients (23 in control group and 23 in experimental group).	To evaluate the effectiveness of home healthcare services for elders post-spinal surgery.	The experimental group demonstrated decreased pain, increased muscle strength, decreased psychological distress, and increased performance of ADLs.
Lim et al^25^	The study utilized a two-level multilevel model to explore factors affecting quality of life.	611 home care users and 20 visiting nurses.	This study explored factors contributing to the quality of life of community-based home visiting care service users.	Enhancing elderly quality of life requires addressing both individual and organizational factors.
Lee and Chang^55^	Nonrandomized study using pre/post design.	94 caregivers of older adults utilizing home healthcare services.	The research aims to improve caregivers' attitudes in home healthcare for older adults through a long-term care education program.	The program significantly improved caregivers' job awareness and safety management. Caregivers' capacity and job satisfaction showed notable improvement after the education program.
Kim et al^38^	Retrospective study of physical medicine and rehabilitation intervention records.	413 users of home mechanical ventilation.	The research aims to describe home mechanical ventilation and the role of physical medicine and rehabilitation in its optimization.	Improved access to home mechanical ventilation can enhance patient outcomes and survival rates, especially in neuromuscular disorders.
Han et al^39^	Retrospective study of medical records.	57 children with neuromuscular disease on home mechanical ventilation.	The research aims to evaluate the application of home mechanical ventilation in children with childhood-onset hereditary neuromuscular diseases.	The application of home mechanical ventilation reduced respiratory morbidity in children with hereditary neuromuscular disease.
Lee et al^42^	Retrospective 1-year cohort study.	22 557 older adults who received nursing home care or home care.	The study aimed to compare ADL outcomes in nursing home care and home care settings for older adults.	Home care is associated with better ADL outcomes and lower costs than nursing home care.
Kang et al^37^	Pre-post survey design.	992 patients with rare and incurable neuromuscular diseases on home mechanical ventilation.	The study aims to identify ventilator usage status in patients with neuromuscular disease requiring respiratory support.	Only 77.2% of patients received follow-up, indicating inadequate respiratory evaluation. Among home-based care patients, 38% lacked regular respiratory evaluation.
Kim and Yeom^50^	Cross-sectional study of convenience sample.	157 family caregivers residing in a single city.	The study aims to examine factors affecting family caregivers' burden and satisfaction related to home care service use.	Home care service use does not significantly reduce caregiver burden or increase satisfaction. Family interactions significantly impact the burden and satisfaction of primary family caregivers.
Kim et al^41^	National claims data analysis using survival analysis.	7841 patients with dementia diagnosed with pressure ulcers before becoming LTCI beneficiaries.	The objective is to assess if long-term care service type affects pressure ulcer incidence among older adults with dementia receiving LTCI.	Institutional care beneficiaries have a higher risk of pressure ulcers compared to home care beneficiaries.
Lee^43^	Retrospective medical record review.	184 patients with pressure ulcers at enrollment for one home care agency.	The study aimed to investigate short-term outcomes of pressure ulcer changes and long-term outcomes of complete healing in patients.	The study found that home care significantly improved pressure ulcer management outcomes in economically vulnerable patients.
Lee and Cho^40^	Secondary data analysis.	7668 older adults with a stroke which resulted in 1099 match pairs using propensity score matching.	The study aims to compare changes in ADLs for older adults with a stroke in home care versus nursing home care.	The study found that older adults with a stroke improved in ADLs and rehabilitation needs with home care, while nursing home care led to deterioration.
Lee et al^34^	Secondary data analysis.	4807 LTCI beneficiaries with pressure ulcers using home-visit nursing services.	The research aims to examine the relationship between home-visit nursing services and hospitalization related to pressure ulcers among beneficiaries in home care settings.	Home-visit nursing services significantly reduced hospitalization risk (OR = 0.68) related to pressure ulcers. Higher ADL scores were positively associated with hospitalization risk (OR = 1.03). Beneficiaries with other nursing care needs had a higher hospitalization risk (OR = 1.37).
Ju et al^58^	A retrospective cohort study.	4173 subjects recommended for home-visit nursing services.	The research aims to evaluate the relationship between home-visit nursing use and hospitalization risk among the elderly.	Home-visit nursing services are essential for healthcare management, especially in the absence of caregivers. Non-use of home-visit nursing increases hospitalization risk among the elderly. Home-visit nursing can help manage chronic diseases and prevent hospitalizations.
Lee et al^49^	Concurrent mixed-method design.	4 patients participating in an individualized HBR program.	The study aims to evaluate the effectiveness of a tailored HBR program for patients with varying social engagement levels.	The tailored HBR program effectively improved physical function in patients with full or partial social engagement. It failed to induce improvements in patients with rare or no social engagement.
Kim et al^45^	Retrospective cohort study.	7112 LTCI beneficiaries aged 60 years or older.	The study aimed to investigate the association between LTC service type and hip fracture incidence among older adults with dementia.	Institutional care beneficiaries had a higher adjusted hazard ratio for hip fractures compared to home care recipients.
Lee et al^56^	In-depth interviews, analyzed using Giorgi’s phenomenological method.	9 family members of recently deceased beneficiaries.	The study aims to understand the experiences of families caring for older adults with non-cancer diseases at the end of their lives.	Caregivers face significant stress due to cognitive impairments and daily life challenges. Educational materials are recommended to support families and healthcare providers in managing caregiving symptoms.
Choi et al^54^	Cross-sectional study design.	74 caregivers of technology dependent children.	The study aimed to examine the living conditions of technology-dependent children and their caregivers at home.	Caregivers reported significant physical, psychological, and financial burdens. Caregivers spent an average of 14.4 hours daily on childcare. Most caregivers found childcare physically burdensome, with over 80% reporting this. The only significant relationship was between economic status and financial burden.
Chin and Lee^35^	Mixed model of repeated measures analysis.	499 frail elderly individuals receiving home healthcare nursing services.	The study aims to evaluate the effects of long-term home health nursing care on frail elderly individuals' health metrics.	The study found significant decreases in systolic and diastolic blood pressure over 8 years among frail elderly individuals receiving home healthcare nursing. Blood glucose levels also showed a gradual decrease, indicating effective long-term management. Cholesterol levels remained stable, with no significant changes observed at follow-up visits.
Lee et al^46^	Retrospective, nonrandomized, nonblinded controlled study.	37 patients who underwent CBCR and HBCR, evaluated at 1 and 6 months.	The study aimed to verify significant differences in cardiopulmonary function between center-based and HBCR programs.	Cardiac rehabilitation significantly improves clinical indicators, reducing all-cause mortality by 20% and heart-disease mortality by 27%. Both CBCR and HBCR programs showed significant improvements in VO2/kg, METs, and EF. The CBCR group exhibited greater improvements in cardiac output compared to the HBCR group.
Cho et al^48^	Non-equivalent control group pre-post-test design.	Convenience sample of 38 participants (18 intervention, 20 control) with first ischemic stroke.	The primary objective is to develop and identify the effectiveness of a transitional intervention for adult stroke patients.	The hospital-to-home transitional intervention improved anxiety, disease severity, health behavior adherence, patient satisfaction, and quality of life in stroke patients. Health behaviors and quality of life scores were significantly higher in the intervention group compared to the control group.
Lee et al^47^	Repeated measures analysis of variance.	135 patients with acute myocardial infarction referred for cardiac rehabilitation.	The study investigates the effectiveness of HBCR on arterial stiffness in patients with acute myocardial infarction.	HBCR effectively lowers arterial stiffness and improves peak oxygen consumption in acute myocardial infarction patients. Significant improvements in pulse wave velocity were observed during the early stages of HBCR. The study suggests that the effectiveness of HBCR may diminish after the initial phase.
Cho and Kim^29^	Structural equation model.	359 people 65 years and older who use home care services.	The study aims to determine the effect of home care service quality on users' voluntary cooperation and participation through self-determination.	The study found that home care quality did not directly affect users' voluntary cooperation or participation in service delivery. Self-determination fully mediated the relationship between service quality and cooperation/participation.
Doan et al^53^	Cross-sectional study design.	165 caregivers of older adults who received home care services.	The study aimed to identify the most difficult decisions faced by family caregivers of older adults receiving home care services.	Family caregivers reported significant decision regret, particularly related to living arrangements and health management decisions. Lower levels of regret were associated with decisions aligning with caregivers' personal preferences. The study found a high average care burden among respondents, indicating substantial emotional and physical strain. Decision conflicts were positively correlated with increased regret, highlighting the complexity of caregiving decisions.
Koo et al^36^	Real-world-based cohort study.	119 patients with type 1 diabetes.	The research aimed to evaluate the effects of the home and self-care program on glycemic control in type 1 diabetes patients.	The home and self-care program effectively improves glycemic control in type 1 diabetes patients, reducing medical costs and enhancing quality of life.
Lee et al^51^	Online survey with statistical analysis.	98 care partners of patients with ALS, with 60.2% being spouses and 34.7% children.	The study aimed to investigate the home care status of care partners of people living with ALS.	Care partners provided an average of 13 hours/day on weekdays and 15 hours/day on weekends. A significant 91.8% of care partners reported experiencing depression, with 28.6% having severe depression. Most care partners preferred to care for patients at home, despite feeling ill-prepared.
**Cost**				
Noh et al^60^	Stepwise multiple linear regression analysis.	89 training hospitals practicing home nursing care.	The study aimed to measure the performance of hospital-based home nursing care and identify affecting factors.	Home nursing care addresses acute hospital bed pressures, supports early discharge, and enhances hospital profitability through revenue diversification.
Hyun et al^61^	Difference-in-difference analysis to evaluate the effects of LTCI program on length of stay.	3 903 448 individuals aged 65 and older.	The research examines the effects of LTCI on the length of stay of senior citizens.	LTCI reduces the length of stay for level 1 and level 2 beneficiaries, but not for level 3. Level 1 and 2 beneficiaries benefit from lower out-of-pocket costs for institutional care services. Level 3 beneficiaries do not experience reduced length of strength due to restrictions on using institutional care services.
Choi et al^57^	Secondary data analysis.	8589 beneficiaries newly enrolled in LTCI program.	The primary objective of the research paper is to explore the impact of in-home service utilization on the institutionalization of older individuals.	The study found that in-home service users had significantly lower rates of institutionalization compared to non-users. Females had a higher rate of institutionalization than males. Dementia increased the likelihood of institutionalization, while cancer decreased it.
Ju et al^58^	A retrospective cohort study.	4173 subjects recommended for home-visit nursing services.	The research aims to evaluate the relationship between home-visit nursing use and hospitalization risk among the elderly.	Home-visit nursing services are essential for healthcare management, especially in the absence of caregivers. Non-use of home-visit nursing increases hospitalization risk among the elderly. Home-visit nursing can help manage chronic diseases and prevent hospitalizations.
Choi et al^62^	Multivariable models to assess the independent effect of implementation of LTCI on medical utilization and medical costs.	3029 beneficiaries who received consecutive LTCI services.	The primary objective is to examine whether LTCI reduces medical utilization and the burden of medical costs for beneficiaries.	LTCI significantly reduced hospitalization rates among beneficiaries compared to non-beneficiaries. Beneficiaries experienced a notable decrease in the burden of medical costs despite rising overall medical expenses for older adults. The introduction of LTCI led to a 46.5% reduction in total average medical expenses for long-term patients. Hospitalization expenses decreased by 148.5%, while outpatient and drug expenses increased marginally.
Boo et al^65^	Secondary data analysis.	30 433 LTCI beneficiaries who died between 2009 and 2013.	The study aims to evaluate medical care use and costs during the last year of life among Korean LTCI beneficiaries from 2009 to 2013.	The study found a decline in older adults dying at home and an increase in deaths in LTC facilities from 2009 to 2013. LTC services did not reduce medical costs by replacing unnecessary inpatient hospitalization.
Lee et al^66^	Cross-sectional study design.	200 noncancer home care nursing patients receiving end-of-life care.	The study aimed to assess end-of-life care needs among non-cancer patients receiving home care nursing who wish to die at home.	The study found that noncancer patients had high care needs for "supporting fundamental needs" when wanting to die at home. Duration of home care nursing was significantly associated with higher end-of-life care needs. Patients with longer home care durations required more comprehensive consideration of their end-of-care needs.
Han et al^59^	Secondary data analysis.	457 524 users and non-users of long-term care services with dementia.	The study aimed to examine the socioeconomic costs of dementia in South Korea based on healthcare and LTC service utilization	LTCI users with home-and community-based services had the lowest annual cost at US $21 391. The highest cost was for non-users admitted to long-term care hospitals, at US $26 978.
Lee et al^46^	Retrospective, nonrandomized, nonblinded controlled study.	37 patients who underwent CBCR and HBCR, evaluated at 1 and 6 months.	The study aimed to verify significant differences in cardiopulmonary function between center-based and HBCR programs.	Cardiac rehabilitation significantly improves clinical indicators, reducing all-cause mortality by 20% and heart-disease mortality by 27%. Both CBCR and HBCR programs showed significant improvements in VO2/kg, METs, and EF. The CBCR group exhibited greater improvements in cardiac output compared to the HBCR group.
Lee and Chin^52^	Secondary data analysis.	650 059 LTCI beneficiaries 60 years or above.	The study aims to identify total care expenditures per older person and related factors affecting these expenditures.	Home care services can delay deterioration in older adults with high care needs. Increased expenditures are linked to older adults who are female, aged 74-84, and have higher care needs. Living alone and having disabilities correlate with lower care service expenditures. Older adults with chronic diseases in residential LTC facilities incur higher costs.
Choi and Yoo^26^	Difference-in-differences technique for analyzing home stay length, costs, and hospitalizations.	538 older adults who use HBPC services.	The study aimed to assess the outcomes of HBPC among older adults.	The study found an increase of 8.3 days in the length of home stay for older adults using HBPC services. Total costs of NHI decreased by US$1241 for older adults using HBPC services compared to the control group. Hospitalization rates decreased to 30.9% after using HBPC services, showing a significant reduction. Admissions to LTC facilities were significantly lower, with a hazard ratio of 0.12 for those using HBPC services.
Choi and Yoo^64^	Difference-in-difference analysis.	17 801 participants and a matched comparison group of 68 145 individuals.	The research aims to evaluate the outcomes of the pilot project for community care among older adults.	The pilot project increased the length of home stay by 4.8 days for participants compared to the comparison group. Participants experienced a reduction of $956 in total costs relative to the matched comparison group.
Lee et al^63^	Retrospective cross-sectional study.	1769 home healthcare patients.	The primary objective is to identify factors affecting the length of stay and discharge destination of home healthcare patients.	The study found significant effects of process variables on length of stay and discharge destination among home healthcare patients. Improved management of HHC quality can reduce unnecessary readmissions and enhance community care efficiency. Patient-specific HHC discharge education can improve service standards and patient satisfaction.

Abbreviations: LTCI, long-term care insurance; ADLs, activities of daily living; OR, odds ratio; HBR, home-based rehabilitation; HBCR, home-based cardiac rehabilitation; HBPC, home-based primary care; CBCR, center-based cardiac rehabilitation; VO2, oxygen consumption; METs, metabolic equivalents; EF, ejection fraction; ALS, amyotrophic lateral sclerosis; LTC, long-term care; NHI, National Health Insurance; HHC, home healthcare.

**Table 2:** Summarized Findings by Dimension (Access, Quality, and Cost)

**Access**
*Caregiver availability is a central determinant of access*: Older adults living with spouses or family caregivers are more likely to use home healthcare services, while those without caregivers or relying on paid caregivers have higher risks of institutionalization.
*Absence of caregivers amplifies risk*: Home-visit nursing becomes especially critical when caregivers are unavailable, underscoring its role as an access bridge for medically fragile populations.
*Home-based services act as a protective factor against institutionalization and hospitalization*: Access to home care and community-based services lowers rates of nursing home admission and hospital use, particularly for older adults with dementia or chronic illness.
*Functional impairment and disease severity increase demand for home-based services*: Individuals who are homebound, have severe disabilities, neuromuscular disease, or lower physical functioning consistently show greater need for home-based primary care, nursing, or mechanical ventilation.
*Gaps in access persist for complex medical needs*: Despite the availability of home mechanical ventilation, a substantial proportion of patients lack regular follow-up and respiratory evaluation, highlighting inequities in service continuity rather than initial access.
*Overall conclusion*: Home healthcare improves access to care for older adults and people with disabilities, but access is uneven and heavily dependent on caregiver presence, disease severity, and system-level follow-up capacity.
**Quality**
*Clinical and functional outcomes are often superior in home care settings*: Studies consistently report improvements in ADLs, pain, muscle strength, cardiopulmonary indicators, glycemic control, blood pressure, and respiratory outcomes among home care recipients compared to institutional care.
*Home care reduces adverse events*: Institutional care is associated with higher risks of pressure ulcers, hip fractures, and functional decline, while home care and home-visit nursing reduce hospitalization and complication rates.
*Rehabilitation delivered at home is effective but context-dependent*:Home-based cardiac and physical rehabilitation improve outcomes, though gains may be smaller than center-based programs or diminish over time without sustained engagement.
*Education and organizational support matter*:Training programs for caregivers and nurses improve safety management, job satisfaction, and care delivery, while self-determination mediates how service quality affects patient engagement.
*Service quality does not automatically translate to caregiver well-being*: Several studies show that while patient outcomes improve, caregiver burden, depression, decision regret, and physical strain remain high—particularly for caregivers of technology-dependent children, patients with ALS, or those at end of life.
*Overall conclusion*: Home healthcare delivers high-quality clinical and functional outcomes and is often safer than institutional care, but quality gains for patients do not consistently reduce caregiver burden without additional psychosocial and educational support.
**Cost**
*Home-based services lower overall medical costs and utilization*: Home care, home-visit nursing, and home-based primary care reduce hospitalizations, length of stay, and admissions to long-term care facilities, leading to net cost savings.
*Cost savings are most evident for lower- to moderate-need populations*: Level 1 and 2 LTCI beneficiaries experience reduced length of stay and out-of-pocket costs, while high-need (Level 3) beneficiaries show fewer cost reductions due to service restrictions.
*End-of-life care presents mixed cost outcomes*: While home care aligns with patient preferences, some studies show that LTC services do not fully substitute for inpatient care at the end of life, limiting cost reductions.
*LTCI plays a major role in cost containment*: Beneficiaries using home- and community-based services consistently incur lower total costs than non-users or those in institutional settings, particularly among people with dementia.
*Hospital-based home nursing supports system sustainability*: Home nursing programs alleviate bed shortages, facilitate early discharge, and diversify hospital revenue streams.
*Overall conclusion*: Home healthcare is largely cost-effective, reducing hospital use and total expenditures, especially when integrated with LTCI and preventive nursing services; however, savings vary by care intensity, disease stage, and end-of-life context.

Abbreviations: LTCI, long-term care insurance; ALS, amyotrophic lateral sclerosis; LTC, long-term care.

###  Home Healthcare Access, Quality, and Cost

 The growing demand for home healthcare services is driven by the increasing prevalence of chronic diseases and the need for long-term care among the elderly. Studies have shown that home healthcare can provide significant benefits in terms of patient outcomes and cost savings.^6,24^ However, challenges related to access, quality, and cost also need to be addressed to fully realize the potential of home healthcare services.^25,26^

####  Access

 Healthcare accessibility in South Korea remains a complex issue influenced by structural, economic, and social determinants. Although the country maintains a well-developed healthcare infrastructure, specific populations, including older adults and individuals with disabilities, frequently encounter substantial barriers to accessing essential healthcare services.^27^ Home-based healthcare has been proposed as a viable strategy to address some of these access-related challenges. However, its success largely depends on various contextual conditions such as policy frameworks, infrastructure support, and caregiver availability.^28,29^

 Introduced in 2008, South Korea’s LTCI program seeks to mitigate healthcare accessibility issues by financing both institutional and home-based care services.^30^ Despite these efforts, informal care delivered by family members often supersedes formal healthcare services due to strong cultural preferences for familial caregiving.^31^ However, this reliance on family members reveals a critical gap; when family support is insufficient or unavailable, individuals often default to institutional care due to limited formal home care services.^31,32^ Research further emphasizes the vulnerability of homebound patients, those with severe disabilities, and individuals dependent on assistance for activities of daily living (ADLs), who face considerable unmet healthcare needs stemming from inadequate transportation options and limited healthcare facility availability in certain regions ^33^. The dependency on family caregivers thus exacerbates these challenges, as the willingness and availability of caregivers strongly determine whether individuals receive adequate home-based care or must resort to institutional long-term care settings.^31-33^

 To address these access challenges, home healthcare services have increasingly focused on bridging geographic and transportation barriers, enabling more timely interventions directly within patients’ homes. Initiatives such as the Patient-Centered Integrated Model of Home Health Care Services in Korea have been developed, emphasizing multidisciplinary collaboration among healthcare providers to enhance service coordination and continuity.^2^ Additionally, integrating remote monitoring technologies has facilitated continuous patient assessment, particularly for chronic diseases such as diabetes and hypertension, improving clinical outcomes and reducing hospital admissions.^26^ Further structured home visit programs conducted by healthcare professionals, including nurses and physicians, have been instrumental in identifying and addressing health issues before they escalate into acute conditions requiring hospitalization.^27^ For instance, home visits have significantly lowered hospitalization rates among elderly patients suffering from pressure ulcers and other chronic conditions, demonstrating the effectiveness of proactive, in-home care.^34^ These comprehensive and integrated approaches are critical to reducing institutionalization, minimizing healthcare disparities, and ultimately enhancing healthcare accessibility for South Korea’s vulnerable populations.

 Across the access-focused studies, caregiver ability and living arrangement consistently emerge as determinants of home-based service use and institutionalization risk. Given that access outcomes were examined in relatively few studies compared with quality and cost, the access synthesis should be interpreted as suggestive rather than definitive. Notably, the access-related findings emphasize continuity and follow-up capacity (eg, regular evaluation) as an important aspect of access beyond initial availability.

####  Quality

 Quality of care in home healthcare services in South Korea encompasses various dimensions, including clinical effectiveness, continuity of care, and patient-centered approaches. As home healthcare services expand, particularly for chronic disease management and post-hospitalization care, maintaining high-quality standards becomes essential for ensuring optimal patient outcomes and satisfaction.^6^

 Research indicates that home healthcare services significantly improve patient outcomes by enabling continuous monitoring and individualized care interventions. For instance, home-based management programs for chronic conditions such as hypertension, diabetes, and cholesterol management have demonstrated substantial improvements in patient health indicators compared to traditional hospital-based outpatient care.^35,36^ Additionally, specialized home healthcare interventions, such as home mechanical ventilation programs, have shown effectiveness in reducing complications and hospitalizations among patients with neuromuscular diseases.^37-39^

 Home healthcare services have also been found to mitigate the deterioration of ADLs more effectively than institutional settings.^40^ Patients receiving home-based care have demonstrated better functional independence, lower rates of severe pressure ulcers, and lower incidence of hip fracture compared to those in nursing homes, indicating superior preventative care and personalized management in home settings.^34,41-45^ Furthermore, structured home-based rehabilitation (HBR) programs contribute significantly to patient recovery and long-term health improvements. Studies have highlighted that the patients engaging in home-based cardiac rehabilitation (HBCR) after myocardial infarction exhibit notable enhancements in cardiopulmonary function, arterial stiffness, and overall physical health. These outcomes are comparable to center-based rehabilitation, suggesting home healthcare’s potential as an effective alternative, especially for patients with limited access to institutional care.^46,47^

 Another important impact includes patient satisfaction and improved quality of life resulting from personalized and continuous care provided by home healthcare services. Patients receiving home healthcare report greater satisfaction due to enhanced nurse-patient relationships continuity of care, and increased autonomy over their health management.^25,29^ Home healthcare interventions, including tailored rehabilitation programs and transitional care following hospitalization, positively impacted on patients’ psychological well-being, adherence to medical recommendations, and overall satisfaction with their healthcare experiences.^48,49^ Additionally, caregiver support, which is a critical component of home healthcare quality, has shown to benefit significantly from structured home healthcare services.^50-52^ Professional home care support has sometimes been linked to reduced caregiver burden, lower emotional distress, and enhanced caregiver satisfaction, as caregivers receive essential training, resources, and opportunities to manage complex patient needs effectively.^48,51,53-56^

 The expansion and structured implementation of home healthcare in South Korea have positively influenced healthcare quality. By integrating multidisciplinary care approaches, remote monitoring technologies, and patient-centered care programs, home healthcare services have improved clinical outcomes, reduced hospital admissions, and significantly enhanced patient and caregiver experiences. These advancements underline the critical role of home healthcare in promoting quality care delivery within South Korea’s evolving healthcare landscape.

 Across studies, patient-centered home-based interventions generally report favorable clinical and functional outcomes, including improvements in ADLs and reduced complications relative to institutional settings. However, the quality evidence is not uniform across outcome domains. Caregiver burden, depression, and decision regret remain substantial in several populations, and some studies report limited improvement in caregiver burden/satisfaction attributable to service use. Together, these findings suggest that patient outcome gains do not consistently coincide with caregiver well-being gains. This pattern indicates heterogeneity in quality depending on whether the outcome is defined at the patient or the caregiver level.

####  Cost

 Cost containment remains a critical consideration in implementing and expanding home healthcare services in South Korea, particularly given the increasing economic pressures posed by an aging population. While home healthcare is often proposed as a more cost-effective alternative to institutional care, evaluating its true economic impacts involves careful consideration of both direct costs and potential savings across the healthcare system.

 Numerous studies indicate that home healthcare can substantially reduce healthcare expenditures by preventing unnecessary hospitalizations and decreasing reliance on costly institutional care.^52,57-59^ Research by Noh et al^60^ demonstrated that hospital-led home healthcare significantly lowered hospitalization rates and healthcare expenses. Similarly, Hyun et al^61^ found that effective home healthcare facilitated earlier hospital discharge, reducing the length of hospital stays and associated costs among older adults.

 The LTCI program, introduced in South Korea back in 2008, has played a pivotal role in mitigating the financial burden of healthcare for beneficiaries by covering home-based care services. LTCI beneficiaries experience notably lower medical costs compared to non-beneficiaries, reflecting substantial financial benefits associated with structured home healthcare support.^52,62,63^ Furthermore, evidence suggests that integrated home healthcare programs combining medical and social services can amplify these cost savings. For instance, participants in community-based home healthcare initiatives reported significant reductions in overall healthcare expenses due to decreased hospital readmissions and extended periods of in-home care.^26,64^

 However, cost-effectiveness in home healthcare is contingent upon the intensity, comprehensiveness, and quality of the services provided. Studies indicated that simply increasing utilization of home healthcare does not always translate directly into cost savings, particularly in end-of-life care contexts where high medical expenditures persist.^65^ Comprehensive and strategically managed home healthcare services, especially those integrative palliative and chronic disease management, are required to realize substantial economic benefits.^66^

 The expansion of home healthcare services in South Korea has positively impacted healthcare costs by reducing avoidable hospitalizations, emergency care utilization, and institutionalization. Programs such as structured HBR and remote chronic disease management have demonstrated notable cost-effectiveness by achieving comparable or improved health outcomes at lower expenses relative to traditional hospital-care.^36,46^ Additionally, pilot initiatives emphasizing integrated care models and caregiver support services have consistently yielded financial benefits by enhancing patient stability, reducing hospital utilization, and promoting efficient healthcare resource use.^26,64^

 Most of the included studies report reductions in utilization (eg, hospitalization, institutionalization, and/or length of stay) associated with home-based or community-based services, often alongside net cost savings. However, cost effects vary by context and intensity of care, with end-of-life studies showing less consistent substitution away from inpatient utilization. Thus, the direction of cost findings is broadly favorable, but the consistency of savings appears contingent on population and care context.

 Overall, home healthcare in South Korea presents a viable economic solution to the rising costs associated with institutional care. By strategically aligning service delivery models with comprehensive care coordination, targeted caregiver support, and technology integration, home healthcare services have shown promise in both enhancing patient outcomes and containing healthcare costs. These advancements underscore the importance of continued investment in structured home healthcare programs to achieve sustainable economic and healthcare system benefits.

## Discussion

 South Korea’s demographic transition, characterized by a rapidly increasing proportion of older adults, presents significant sustainability challenges for its healthcare system, which historically relies heavily on hospital-based care models. Institutional care is becoming increasingly unsustainable due to rising healthcare expenditures, overcrowding of acute care facilities, and a growing preference among elderly individuals to age comfortably within their homes.^67^ Aligning with the iron triangle framework, home healthcare emerges as a potentially effective strategy to simultaneously enhance healthcare access, maintain or improve quality of care, and control healthcare expenditures.

 The findings from this review highlight critical opportunities and persistent barriers in implementing home healthcare in South Korea. Home-based healthcare services have demonstrated notable potential for improving patient outcomes, particularly among populations vulnerable to institutionalization, including older adults and individuals with severe disabilities. Structured home health initiatives, such as multidisciplinary care models and remote monitoring technologies, have shown efficacy in enhancing clinical outcomes and patient satisfaction by enabling timely interventions and continuous health management directly with the patient’s home environment.^2,7^ Furthermore, targeted caregiver support initiatives have significantly alleviated caregiver burden and improved overall healthcare quality, reinforcing the importance of integrating caregiver-focused strategies within home healthcare programs.^51^

 Despite these advancements, the review identified substantial challenges limiting the scalability and effectiveness of home healthcare in South Korea. Structural barriers, particularly inadequate infrastructure and uneven geographic distribution of healthcare resources, continue to constrain access to home healthcare services, especially in rural and underserved regions. For example, while remote monitoring and telehealth technologies offer significant potential to enhance continuity and efficacy of home healthcare, their real-world adoption among older adults in South Korea is constrained by digital literacy gaps, limited device accessibility, and low technological self-efficacy among elderly populations. These barriers risk exacerbating existing access inequities, particularly for socioeconomically vulnerable and rural older adults. Moreover, South Korea’s historically restrictive telehealth regulatory framework, including limitations on direct physician-to-patient telemedicine, has further impended full integration of digital health into routine home care delivery. Additionally, the persistent reliance on informal caregiving underscores the need for systemic improvements in formal caregiver training and professional support structures. To ensure the long-term sustainability of home healthcare in South Korea, policy responses must extend beyond caregiver education to include systemic financial and workforce supports for family caregivers. These should include direct financial subsidies or wage-replacement stipends for family caregivers, expansion of publicly funded professional respite services, and formal integration of family caregiving into LTCI reimbursement frameworks. In parallel, the limited role of physicians in routine home visits represents a structural workforce constraint that places an upper bound on the clinical acuity that can be safely managed in the home setting. Without addressing these systemic deficiencies, the effectiveness and sustainability of home healthcare as an alternative to institutional care remains at risk.^6,30^

**Figure 2:**
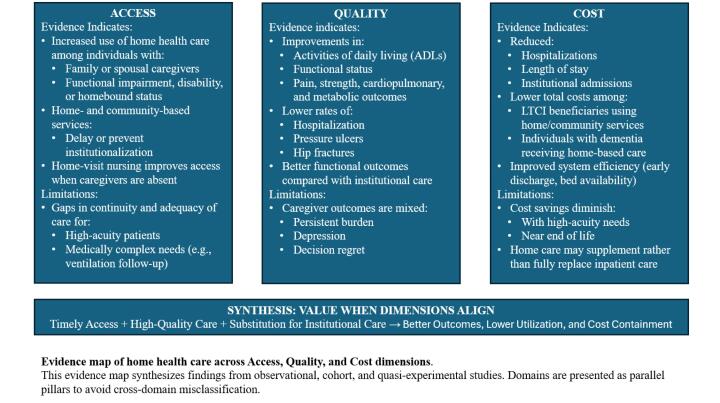
Evidence Map of Findings. Abbreviations: LTCI, long-term care insurance; ADLs, activities of daily living.

 Economic sustainability remains another critical factor influencing the adoption and expansion of home healthcare services. The reviews found consistent evidence supporting the cost-saving potential of home healthcare through reduced hospitalization rates and decreased reliance on emergency care and institutionalization. However, achieving these economic benefits depends significantly on the comprehensiveness and management of home healthcare services. Programs that strategically integrate medical, social, and technological elements, such as community-based home health initiatives and structured rehabilitation services, have demonstrated substantial cost efficiencies and improved patient outcomes.^26,66^ Nevertheless, the financial viability of home healthcare also hinges on appropriate reimbursement models and public funding structures that align provider incentives with high-quality, cost-effective care (Figure 2).

 Placing South Korea’s home healthcare model within an international context further clarifies both its constraints and opportunities for reform. In Japan, physician-led home visits are formally integrated into the national insurance system, enabling management of higher-acuity patients and reducing unnecessary hospitalizations,^68^ while the United Kingdom’s community-based primary care infrastructure embeds general practitioners directly into home-based care pathways.^69^ In the United States, programs such as hospital-at-home and home-based primary care explicitly expand physician oversight into the home setting for medically complex patients, supported by value-based reimbursement models.^70^ These international experiences suggest that stronger physician integration, aligned reimbursement mechanisms, and formalized escalation pathways are critical policy levers for expanding the clinical scope and continuity of home healthcare and offer relevant design lessons for strengthening South Korea’s evolving model.

 Future policy directions should prioritize expanding infrastructure for home healthcare delivery, particularly in rural and underserved regions, to reduce geographic disparities. Investment in comprehensive caregiver education and ongoing professional support is necessary to ensure consistent quality and effective service delivery. Moreover, leveraging advanced technologies for remote monitoring and telehealth can enhance service efficiency, improve patient outcomes, and reduce costs. Continued research is required to optimize home healthcare service models, evaluate the sustainability of various care coordination strategies, and further explore home healthcare’s long-term impacts on patient outcomes and systemic healthcare expenditures.

## Limitations and Future Research

 There are several limitations inherent in the study. Selection bias is a concern when there is subjectivity involved in the screening and selection process. The search syntax employed attempted to capture the largest number of relevant documents but may not have captured all available documents. Publication bias exists as the initial search was limited to research databases and these databases restrict the publications searched, the years searched, and only capture published documents and publication is often based upon the significance of the findings. To mitigate this limitation, the searches were not limited to academic journals or peer reviewed articles but also included books, book chapters, trade/industry journals, newspapers, news wire releases, and gray literature. Additionally, restraining the dates searched may have also limited the studies identified. Lastly, the exclusive focus on the South Korean healthcare system, which may constrain the external generalizability and transferability of the findings to other national contexts, is an additional limitation of this study. South Korea’s unique combination of universal health insurance, LTCI, rapid population aging, and strong cultural norms surrounding family caregiving shapes both the structure and functioning of home healthcare in ways that may differ substantially from systems in various parts of the world.

 Future research should explore how different models of home healthcare impact both patient outcomes and healthcare system sustainability. As South Korea continues to navigate the challenges of an aging society, optimizing home healthcare utilization will be critical in ensuring both economic efficiency and quality of life for older adults.

## Conclusions and Relevance

 Home healthcare in South Korea holds a significant potential to address the challenges posed by the country’s aging population by improving healthcare access, maintaining quality, and reducing costs. However, its integration into the broader healthcare system remains limited due to regulatory, financial, and structural barriers. This review highlights the need for standardized protocols, expanded provider participation, and policy reforms that promote sustainable funding mechanisms and workforce development. Moving forward, a more integrated approach that aligns home healthcare with primary and specialty care services will be essential to ensuring it scalability and long-term viability. With targeted reforms and investments, home healthcare can play a critical role in enhancing patient-centered care and relieving strain on institutional healthcare facilities, ultimately contributing to a more sustainable and equitable healthcare system in South Korea.

## Disclosure of artificial intelligence (AI) use

 Not applicable.

## Ethical issues

 Not applicable.

## Conflicts of interest

 Authors declare that they have no conflicts of interest.
